# Annealing Behavior of a Mg-Y-Zn-Al Alloy Processed by Rapidly Solidified Ribbon Consolidation

**DOI:** 10.3390/ma17184511

**Published:** 2024-09-13

**Authors:** Jenő Gubicza, Kristián Máthis, Péter Nagy, Péter Jenei, Zoltán Hegedűs, Andrea Farkas, Jozef Vesely, Shin-ichi Inoue, Daria Drozdenko, Yoshihito Kawamura

**Affiliations:** 1 Department of Materials Physics, Faculty of Science, ELTE Eötvös Loránd University, Pázmány P. sétány 1/A, H-1117 Budapest, Hungary; nagyp@student.elte.hu (P.N.); jenei.peter@ttk.elte.hu (P.J.); 2 Department of Physics of Materials, Faculty of Mathematics and Physics, Charles University, Ke Karlovu 5, 12116 Prague, Czech Republic; kristian.mathis@matfyz.cuni.cz (K.M.); aszabo.cuni@gmail.com (A.F.); vesely@gjh.sk (J.V.); daria.drozdenko@matfyz.cuni.cz (D.D.); 3 Deutsches Elektronen-Synchrotron DESY, Notkestr. 85, 22603 Hamburg, Germany; 4 Magnesium Research Center, Kumamoto University, 2-39-1 Kurokami, Chuo-ku, Kumamoto 860-8555, Japan; shinoue7@kumamoto-u.ac.jp (S.-i.I.); rivervil@gpo.kumamoto-u.ac.jp (Y.K.)

**Keywords:** Mg-Zn-Y-Al alloy, long-period stacking ordered (LPSO) phase, cluster-arranged layers (CALs), annealing, lattice constant, thermal expansion coefficient

## Abstract

Mg-Y-Zn-Al alloys processed by the rapidly solidified ribbon consolidation (RSRC) technique are candidate materials for structural applications due to their improved mechanical performance. Their outstanding mechanical strength is attributed to solute-enriched stacking faults (SESFs), which can form cluster-arranged layers (CALs) and cluster-arranged nanoplates (CANaPs) or complete the long-period stacking ordered (LPSO) phase. The thermal stability of these solute arrangements strongly influences mechanical performance at elevated temperatures. In this study, an RSRC-processed Mg—0.9%, Zn—2.05%, Y—0.15% Al (at%) alloy was heated at a rate of 0.666 K/s up to 833 K, a temperature very close to melting point. During annealing, in situ X-ray diffraction (XRD) measurements were performed using synchrotron radiation in order to monitor changes in the structure. These in situ XRD experiments were completed with ex situ electron microscopy investigations before and after annealing. At 753 K and above, the ratio of the matrix lattice constants, *c*/*a*, decreased considerably, which was restored during cooling. This decrease in *c*/*a* could be attributed to partial melting in the volumes with high solute contents, causing a change in the chemical composition of the remaining solid material. In addition, the XRD intensity of the secondary phase increased at the beginning of cooling and then remained unchanged, which was attributed to a long-range ordering of the solute-enriched phase. Both the matrix grains and the solute-enriched particles were coarsened during the heat treatment, as revealed by electron microscopy.

## 1. Introduction

In recent decades, a new class of Mg alloys has been introduced, which contain a low amount (1–2 at%) of transition metals (TMs, e.g., Zn, Cu, or Ni) and rare-earth metals (REs), such as Y or Gd. The alloying elements together with Mg form a long-period stacking ordered (LPSO) structure as a secondary phase [1,2,3,4,5]. These Mg alloys with LPSO structures exhibit an excellent mechanical strength [1,6,7,8], establishing interest in these compositions. For instance, Mg-Y-Zn alloys containing an LPSO phase and processed by rapidly solidified powder metallurgy exhibit a yield strength higher than 600 MPa, with an acceptable elongation to failure of about 5% [1]. The application of the rapidly solidified ribbon consolidation (RSRC) technique to dilute Mg-Zn-Y alloys resulted in a yield strength over 350 MPa and an elongation of about 20% [9]. Concerning the formation of the LPSO structure, ab initio molecular dynamic simulations revealed that, in Mg-Zn-Y alloys, the solute atoms cause lattice distortion, which induces the rearrangement of the neighboring Mg atoms into a local face-centered cubic (FCC) configuration in the hexagonal close-packed (HCP) structure [10]. This HCP-to-FCC transition is usually restricted to only four basal planes, yielding the sequence A’B’C’A’ instead of ABAB, where B’ and C’ are layers decorated with both solute Zn and Y atoms, while A’ contains Y solutes beside Mg atoms. The B’-C’ pair of solute-enriched layers is referred to as a solute-enriched stacking fault (SESF), since the A’B’C’A’ close-packed plane sequence can also be considered as an HCP structure containing a stacking fault (SF). A block of four basal planes with the stacking of A’B’C’A’ is called a cluster-arranged layer (CAL) [10]. Its formation mechanism has also been validated by high-resolution STEM and in situ synchrotron XRD [11,12]. Former studies [10,13,14] have shown that, in Mg-Zn-Y alloys, the solutes are mostly clustered at individual CALs. In addition, if CALs are arranged into a long-range periodic structure, an LPSO phase forms in the Mg matrix. In 10H, 18R, 14H, and 24R LPSO structures, the neighboring CALs are separated by one, two, three, and four pure Mg basal planes, respectively. If there is no long-range periodicity in the variation of CALs, i.e., the number of basal planes between the consecutive CALs is not consistent, but rather varies between one and four, the structure is referred to as a cluster-arranged nanoplate (CANaP) [15].

The thermal stability of the CAL, CANaP, and LPSO structures is important considering the high-temperature applications of these dilute Mg-TM-RE alloys. It has been shown by thermodynamic calculations that the 18R LPSO structure is a high-temperature phase which is stable above 756 K [16]; therefore, it can form directly from the melt during casting of Mg-TM-RE alloys. On the other hand, the 14H LPSO phase is stable below 810 K, thus, it is formed rather by a solid-state transformation [16]. In the temperature range between 756 and 810 K, the two LPSO phases can co-exist in equilibrium. Accordingly, it was reported that the 18R structure in as-processed Mg alloys was transformed into the 14H LPSO phase applying isothermal heat treatments for hours at temperatures between 623 and 773 K [4,17]. Contrary to the prediction of the thermodynamic calculations, the coexistence of 14H and 18R structures in LPSO particles has been observed at room temperature (RT), e.g., in a Mg_97_Zn_1_Y_2_ alloy processed by the RSRC technique and subsequently annealed at 673 K for 48 h [13]. Moreover, the thermal behavior of an 18R LPSO Mg_85_Zn_6_Y_9_ polycrystal processed by directional solidification was studied in situ by synchrotron radiation in the temperature range between 95 and 440 K [18]. It was found that the thermal expansion coefficients in both directions, *a* and *c*, were lower for the 18R LPSO phase (1.8–1.9 × 10^−5^ K^−1^) than for pure Mg (2.5–2.6 × 10^−5^ K^−1^). However, an in-depth study of the thermal behavior of RSRC-processed Mg-TM-RE alloys is missing from the literature.

The present study investigates the microstructure changes in an RSRC-processed Mg—0.9%, Zn—2.05%, Y—0.15% Al (at%) alloy during annealing up to a temperature close to melting point (833 K). The evolution of the matrix and the secondary phase is studied by in situ X-ray diffraction (XRD) using synchrotron radiation. In addition, the microstructure before and after the heat treatment is investigated using microscopic techniques. This study contributes to the assessment of the thermal stability of Mg alloys with solute-enriched CANaPs and LPSO structures.

## 2. Materials and Methods

### 2.1. Processing of the Material

The initial Mg—0.9, Zn—2.05, Y—0.15 Al (at%) alloy was produced by casting under an Ar atmosphere in a high-frequency induction furnace using pure Mg (99.99 wt%), Y (99.5 wt%), Zn (99.9 wt%), and Al (99.99 wt%). The melting was carried out in a cylindrical carbon crucible. Rapidly solidified ribbons were prepared from the as-cast alloy by a single-roller liquid quenching melt-spinning method [19] using a rotating copper disc at a roll peripheral speed of 10 m/s and a cooling rate of 1.8 × 10^4^ K/s. The rapidly solidified ribbons were put into Cu billets and degassed at 523 K for 15 min. Then, the billets were subjected to a pre-extrusion heat treatment at 698 K for 24 h. The heat-treated billets were extruded at 623 K with an extrusion ratio of R10 and a ram speed of 2.5 mm/s.

### 2.2. In Situ XRD Study of the Structure during Annealing

The evolution of the structure of the alloy during annealing was monitored in situ using synchrotron XRD at the Deutsches Elektronen-Synchrotron (DESY) facility, Hamburg, Germany. The XRD experiment was performed in transmission mode with a beam energy of 82.5 keV, corresponding to an X-ray wavelength of λ = 0.015028 nm. The size of the beam was 150 × 150 μm^2^. The sample was heated from RT (293 K) to 833 K (560 °C) at a rate of 0.666 K/s, and then cooled down to RT. Our intention was to keep the temperature interval between the successive diffractograms at 1 K. Considering the acquisition time of an XRD pattern (1.5 s), this condition yielded a heating/cooling rate of 0.666 K/s. Cooling was performed at the same rate as heating; however, below 433 K, the cooling rate was gradually reduced in order to achieve a smooth approach to RT. During the test, two-dimensional XRD patterns were collected in situ using a Pilatus CdTe 2M detector, while the sample to detector distance was 892 mm. A LaB_6_ standard sample (NIST SRM 660c) was used to calibrate the detector position and measure the instrumental broadening of the setup. The detector images were reduced to conventional diffractograms using pyFAI [20]. In this process, the intensity was integrated along the whole Debye–Scherrer rings in the 2D patterns.

The lattice constants of the Mg matrix were determined from the XRD peak positions. First, the values of the interplanar spacing (*d*) were determined from the Bragg angle (*θ*) using the Bragg equation for fifteen peaks in the *2θ* range between 3 and 8.5° [21]. Then, the hexagonal lattice constants, *a* and *c*, of the Mg matrix were obtained by minimizing the sum of the squares of the differences between the experimentally observed interplanar spacing and the values calculated from the following formula [21]:

(1) $$d={\left[\frac{4}{3a^{2}}\left(h^{2}+k^{2}+hk\right)+\frac{l^{2}}{c^{2}}\right]}^{-1/2},$$

where *h*, *k*, and *l* are the three reflection indices.

### 2.3. Microstructure Study before and after Annealing Using Electron Microscopy Methods

Scanning electron microscopy (SEM) was employed for the investigation of the microstructure before and after annealing using a Zeiss CrossBeam Auriga microscope, equipped with an EDAX Velocity high-speed acquisition camera and a backscattered electron (BSE) detector. For microscopic observations, the surfaces of the samples were mechanically ground and polished using diamond pastes down to a 1 µm particle size. Furthermore, the surface was additionally ion beam polished by Ar ions using a Leica EM RES102 system (Wetzlar, Germany) to provide high-quality electron back-scattered diffraction (EBSD) images. The EBSD data were analyzed using TSL OIM Analysis software version 8 (TexSem Laboratories, Provo, UR, USA). Transmission and scanning transmission electron microscopy (TEM and STEM, respectively) studies were performed using a JEOL 2200 FS microscope (Akishima, Japan) operated at 200 kV. The foils prepared for TEM investigations were first mechanically polished, and then the final thinning was performed using the ion milling system Leica EM RES102.

## 3. Results

### 3.1. Annealing Behavior of the Matrix as Determined by In Situ XRD

**Figure 1:**
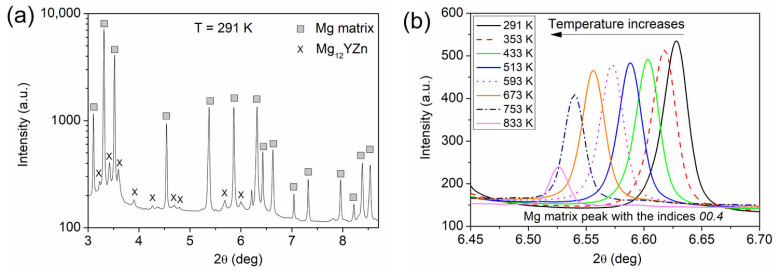
(**a**) XRD pattern taken on the initial sample before heat treatment. The intensity is plotted in logarithmic scale. (**b**) Change om the peak position for reflection 00.4 of the matrix with increasing the temperature.

Figure 1a shows the XRD pattern taken on the initial sample before heat treatment. The strong diffraction peaks correspond to an α-Mg matrix, while the weak peaks marked by letter X indicate the existence of a secondary phase. The peak positions of the secondary phase resemble those of an 18R LPSO structure with the composition of Mg_12_YZn and supercell lattice constants of *a* = 1.12 nm and *c* = 4.70 nm [22,23,24,25]. On the other hand, the STEM investigation presented later in this study reveals that the structure of the secondary phase particles is not uniform, i.e., different LPSO phases, such as 18R and 14H, may coexist. Therefore, the peaks of the secondary phase were not indexed and the evolution of the lattice constants of the LPSO phase during annealing was not investigated. During annealing, the formation of additional phases was not observed by XRD up to the highest applied temperature of 833 K. It is noted that, due to the low wavelength of the synchrotron X-rays used, the diffraction peaks of the studied crystalline phases appeared at low scattering angles. When increasing the temperature during heating, the XRD peaks of both the matrix and the secondary phase were shifted to lower diffraction angles due to thermal expansion. As an example, Figure 1b shows the change in the peak position for reflection 00.4 of the Mg matrix. The decrease in the peak intensity for higher temperatures can be attributed to the Debye–Waller factor [21]. In addition, the partial melting of the sample at 833 K may also contribute to the strong reduction in the XRD peak intensity between 753 and 833 K, as will be discussed later.

**Figure 2:**
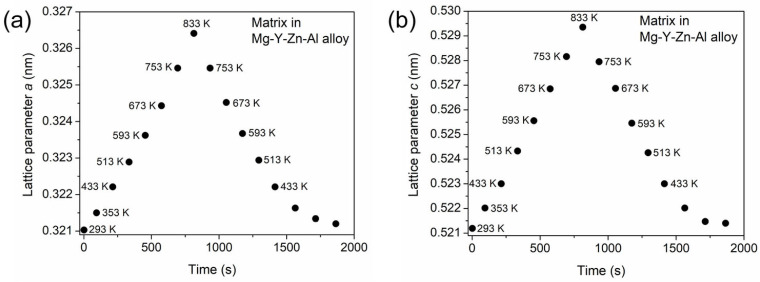
The change in the lattice constants *a* and *c* of the matrix, respectively, as a function of the measuring time (shown in (**a**) and (**b**), respectively). The corresponding temperatures are indicated for those data points which were obtained at constant heating/cooling rate. The error of the data is represented by the size of the symbols.

Figure 2a,b show the changes in the lattice constants *a* and *c* of the matrix, respectively, as a function of the measuring time. The relative error of the lattice parameters was about 10^−4^, which approximately corresponds to the size of the symbols in Figure 2. This uncertainty was estimated from the difference between the measured and the calculated values of the interplanar spacings. The latter was obtained from Equation (1) using the lattice constant values determined by the method described in Section 2.2. It should be noted that, although diffractograms were detected for every Kelvin degree between RT and 833 K, the lattice constants and the phase fraction were determined for far fewer temperatures, since the preliminary qualitative inspection of the XRD patterns suggested a monotonous trend in the position and the area of the peaks. Figure 2 reveals that the variation in the lattice parameters was not symmetrical at the end of cooling compared to the beginning of heating, which can be explained by the change in the cooling rate when the temperature approached RT. In this study, the temperature dependence of the lattice constants was analyzed only for the heating stage.

**Figure 3:**
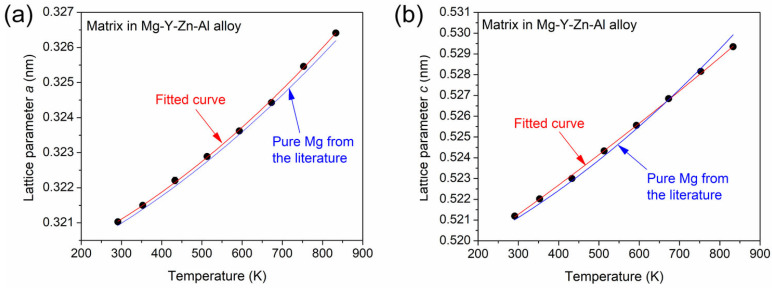
The lattice parameters *a* and *c* of the matrix versus the temperature during heating (shown in (**a**) and (**b**), respectively). The error of the data is represented by the size of the symbols. The red curves correspond to quadratic polynomials fitted to the data points. The blue lines indicate the lattice constant values versus the temperature for pure Mg taken from Ref. [26].

Figure 3a,b show the lattice parameters *a* and *c* of the matrix, respectively, versus the temperature during heating. The error of the data is represented by the size of the solid circle symbols. During cooling, the lattice constant varied reversibly until the cooling rate was constant. The data points were fitted by a quadratic polynomial in order to determine the relationship between the lattice constants and the temperature (indicated by red lines in Figure 3). The following equations were obtained for the matrix lattice parameters *a* and *c* expressed in nm, respectively:

(2) $$a=0.3194+4.03\times 10^{-6}\times T+5.22\times 10^{-9}\times T^{2},$$

(3) $$c=0.5173+1.24\times 10^{-5}\times T+2.44\times 10^{-9}\times T^{2},$$

where *T* is the temperature in Kelvin degrees. The relative error of the coefficients of the 0th-order terms in Equations (2) and (3) is about 0.05%, while for the 1st- and 2nd-order terms, the uncertainty is 5–10%. These error values were given by the software used for fitting the lattice constant versus temperature data. Figure 3a shows that the lattice constant *a* increased with an increasing temperature in a similar way to that formerly observed for pure Mg (indicated by the blue line) [26]. The consistently slightly higher *a* values for the presently studied matrix can be attributed to the solute elements. The same is valid for *c*, except at the two highest applied temperatures (753 and 833 K), where the lattice constant in direction *c* was lower than that for pure Mg. This change will be discussed in Section 4.

**Figure 4:**
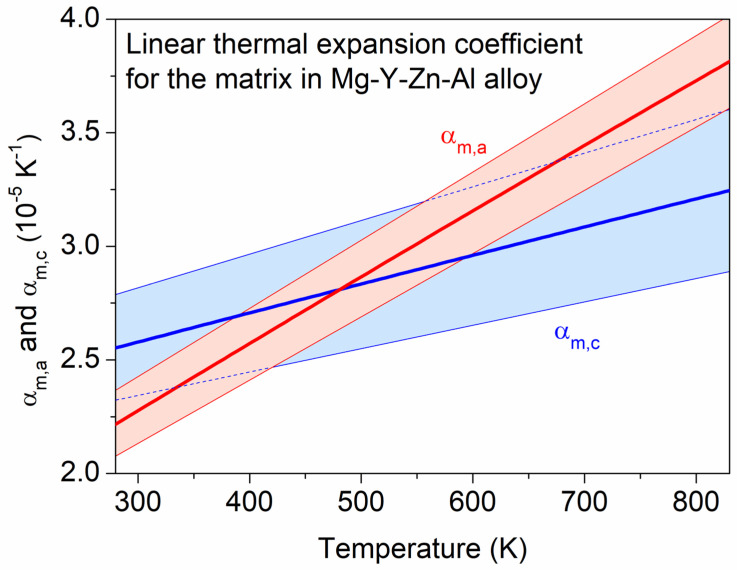
The linear thermal expansion coefficients of the matrix in directions *a* and *c* (denoted as $\alpha_{m,a}$ and $\alpha_{m,c}$, respectively) versus the temperature. The strips around these curves illustrate the uncertainty of these relationships as estimated from the error of the fitting on the lattice constant versus temperature data.

The linear thermal expansion coefficient of the matrix in direction *a* ($\alpha_{m,a}$) was determined as the ratio of the derivative of the *a* (*T*) function given in Equation (2) with respect to the temperature over *a* (*T*). A similar calculation was applied for the determination of the thermal expansion coefficient in direction *c* ($\alpha_{m,c}$) using the derivative of Equation (3). The obtained $\alpha_{m,a}$ and $\alpha_{m,c}$ versus *T* functions are plotted with red and blue lines, respectively, in Figure 4. The strips around these curves illustrate the uncertainty of the thermal expansion coefficient values, which was estimated from the errors of the parameters of Equations (2) and (3) considering the rules of error propagation. Both linear thermal expansion coefficients increased when increasing the temperature in the range between 293 and 833 K. This trend is in line with that formerly observed for pure Mg [26]. In addition, the values of $\alpha_{m,a}$ and $\alpha_{m,c}$ at RT determined in the present study (2.3 × 10^−5^ K^−1^ and 2.6 × 10^−5^ K^−1^, respectively) are close to the values obtained for pure α-Mg (2.2 × 10^−5^ K^−1^ and 2.6 × 10^−5^ K^−1^ for $\alpha_{m,a}$ and $\alpha_{m,c}$, respectively) [18]. Considering the uncertainty of the thermal expansion coefficients, the temperature dependence of $\alpha_{m,a}$ and $\alpha_{m,c}$ is only slightly different.

**Figure 5:**
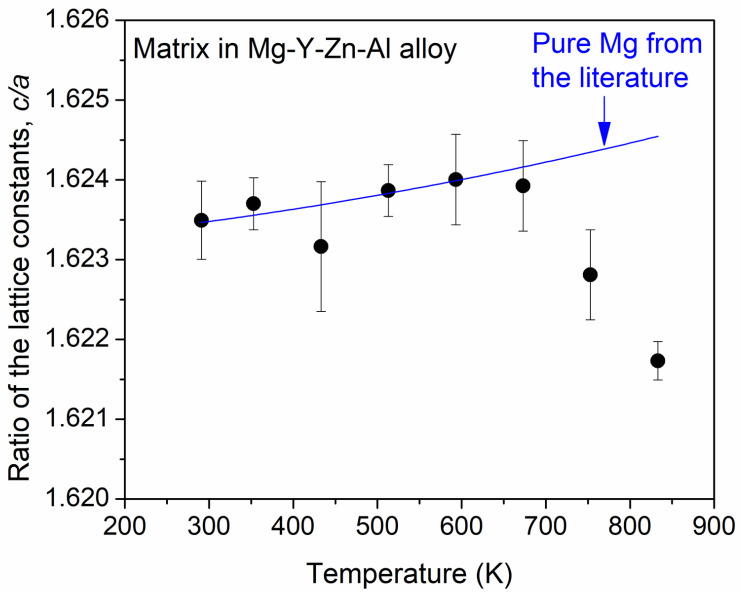
Ratio of the lattice constants *c* and *a* of the matrix versus the temperature. The blue curve indicates the trend for pure Mg observed formerly in Ref. [26].

The ratio of the lattice constants *c* and *a* of the matrix versus the temperature is plotted in Figure 5. Up to the temperature of 673 K, the trend follows that formerly observed for pure Mg, i.e., it increased slightly from about 1.6235 to 1.6240, as shown by the blue curve [26]. On the other hand, at 753, K the *c*/*a* ratio starts to decrease, and this trend continues when the temperature increases up to 833 K. At this maximum temperature, the *c*/*a* ratio is reduced to ~1.6217. The lower values of the *c*/*a* ratio at 753 and 833 K can be explained by the reduced rate of the increase in the lattice parameter *c* at high temperatures compared to pure Mg (see Figure 3b).

### 3.2. Change in the Amount of the Secondary Phase Obtained by Synchrotron XRD

Figure 1a shows that the weak secondary phase peaks overlap with the strong reflections of the matrix. In addition, due to the coexistence of different LPSO structures in the secondary-phase particles (as will be revealed by STEM in the next section), the peaks were not indexed and the lattice parameters were not determined from the secondary phase peak positions. On the other hand, the variation in the amount of the secondary phase during annealing was calculated from the change in the peak intensity, as described in the next paragraph. The intensity of the XRD reflections was determined as the area under the peak after background subtraction. The XRD peak area is influenced by the set of the background; therefore, the error of the peak intensity was estimated from the variation in the peak area when the background subtraction was repeated.

**Figure 6:**
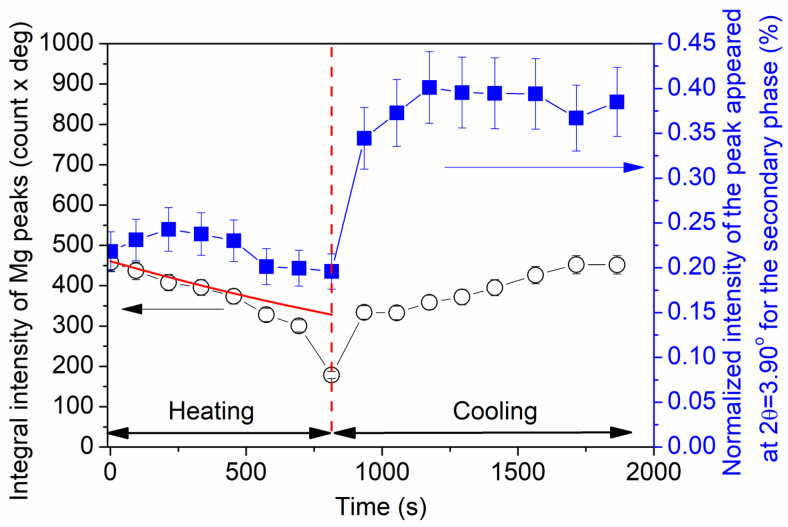
Sum of the areas under the Mg matrix reflections (integrated intensity) versus the time of annealing (open circles). The red line represents the reduction in the intensity during heating caused by the Debye–Waller factor as determined formerly for Mg [27]. The secondary-phase fraction evolution is characterized by the change in the area under the XRD peak appearing at 2θ = 3.90° normalized by the sum of the areas under the matrix reflections (indicated by blue squares).

The evolution in the amount of the solute-enriched secondary phase was characterized with the variation in the area under the XRD peak at 2θ = 3.90° during heating and cooling. This reflection was selected since it is far from the large peaks of the matrix; therefore, the area under this reflection is trustworthy. It should be noted, however, that the peak intensity varies when the temperature changes, even if the phase fraction remains constant. A decrease in the peak intensity when increasing the temperature is caused by the thermal vibration of the atoms at the lattice points, an effect usually taken into account by the Debye–Waller factor [21]. Indeed, the sum of the areas under the Mg matrix reflections was reduced when increasing the temperature, as shown by the open circles in Figure 6. The red line represents the reduction in the intensity during heating caused by the Debye–Waller factor, as formerly determined for Mg [27]. A slight deviation between the experimental Mg integral intensity and that predicted by the Debye–Waller factor was observed for the sixth and seventh measurement points during heating, corresponding to temperatures of 473 and 753 K. On the other hand, a large deviation was detected for the last point at 833 K. At these temperatures, in addition to the effect of the Debye–Waller factor, most probably a partial melting of the material contributed to the decrease in the intensity of the Mg matrix peaks. These two effects also influenced the intensity of the secondary-phase reflections. Therefore, the secondary-phase fraction is described with the area under the XRD peak, which appeared at 2θ = 3.90°, normalized by the sum of the areas under the matrix reflections. The variation in this normalized intensity versus the time of annealing is shown by blue squares in Figure 6. During heating, the amount of the secondary phase did not change considerably. On the other hand, at the beginning of the cooling process, the XRD intensity of the secondary phase was doubled and then remained practically constant until the end of the heat treatment. This observation is valid not only for the secondary phase peak appearing at 2θ = 3.90°, but also for the other reflections, so this was not a result of the change in the crystallographic texture. The described increase may occur by either joining the solute atoms to the secondary-phase particles or increasing the long-range order in the LPSO structure. Nevertheless, our XRD study suggested a pronounced change in the secondary phase; therefore, additional direct observations of the microstructure before and after the heat treatment were performed by microscopic techniques, and the results are presented in the next section.

### 3.3. Comparison of the Microstructure before and after Annealing Using Microscopy

**Figure 7:**
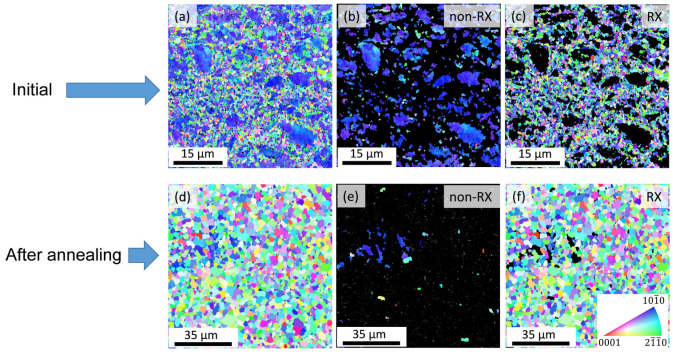
EBSD grain orientation maps of the matrix for the initial material (**a**–**c**) and the sample after annealing (**d**–**f**). Entire microstructure (**a**,**d**), non-recrystallized (non-RX) regions (**b**,**e**), and recrystallized (RX) areas (**c**,**f**).

Figure 7 presents the microstructures before and after the heat treatment via EBSD grain orientation maps. Alongside the entire orientation map (Figure 7a), maps for the non-recrystallized (non-RX) and recrystallized (RX) grains are presented separately in Figure 7b,c, respectively, for the initial material. The same maps after annealing are shown in Figure 7d–f. In the latter case, the RX fraction includes the crystallites solidified from the partially melted volumes at the beginning of cooling. The grains with a grain orientation spread (GOS) lower than 2° were considered as RX regions. The fraction of the RX region and the average grain sizes in the RX area for the initial and annealed materials are listed in Table 1. In the initial sample, the RX fraction was 62%, which increased to 97% during annealing, while the average size of the RX grains increased from 0.63 to 2.80 μm. The average size of the non-RX grains was about 2.3 μm in the initial material; however, its change during annealing could not be determined with sufficient certainty due to the low number of non-RX grains (see Figure 7e).

**Figure 8:**
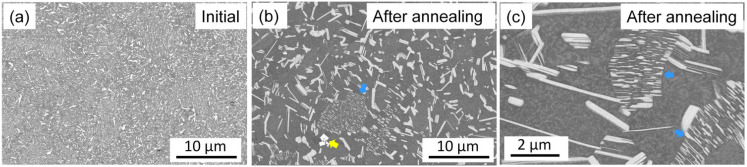
SEM-BSE images taken on the initial sample (**a**) and the material after annealing (**b**,**c**). The yellow arrow indicates YH_2_ particles. The blue arrows indicate non-RX grains containing fine solute-enriched secondary-phase particles.

**Figure 9:**
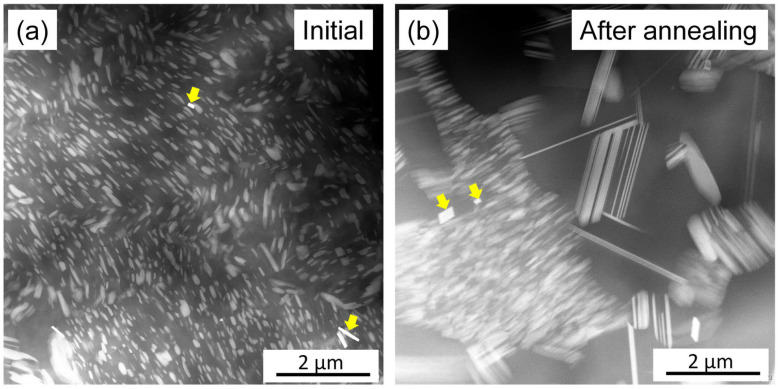
STEM images obtained on the initial specimen (**a**) and the alloy after annealing (**b**). The yellow arrows indicate YH_2_ particles.

The solute-enriched secondary phases in the initial and annealed samples show a bright contrast in the SEM-BSE images in Figure 8. The comparison of these micrographs suggests that the fine secondary-phase particles in the initial material were transformed into coarser ones in the majority of the alloy during annealing. The coarsening of the solute-enriched particles was in accordance with the decrease in the XRD peak width. It should be noted, however, that in some α-Mg matrix grains (indicated by blue arrow in Figure 8b), the solute-enriched particles remained similarly fine as in the initial material. For instance, Figure 8c shows a BSE image with a higher magnification, indicating fine precipitates in two grains (highlighted by blue arrows). Most likely, the alloying elements formed larger secondary-phase particles during the nucleation of the RX grains, while a fine solute-enriched structure remained in the non-RX matrix grains. Figure 8b illustrates the existence of very bright rectangles (indicated by yellow arrow), which are YH_2_ particles, as proven by TEM diffraction. This phase was also detected in the initial alloy, as shown in the STEM micrograph in Figure 9a.

Figure 9a,b show STEM images taken on the initial and the annealed samples, respectively. Again, the bright contrast indicates solute-enriched precipitates. In the initial sample, the secondary-phase particles are fine (less than 1 μm in size) and distributed homogeneously (see Figure 9a). In the micrograph taken after annealing, large precipitates can be seen (see right side of Figure 9b), while in the grain at the left side of the image, the secondary-phase particles remain fine (see Figure 9b). In both conditions, besides the long secondary-phase lamellas, small YH_2_ particles were observed, as indicated by the yellow arrows in Figure 9. The size of the YH_2_ particles was not influenced by the heat treatment.

**Figure 10:**
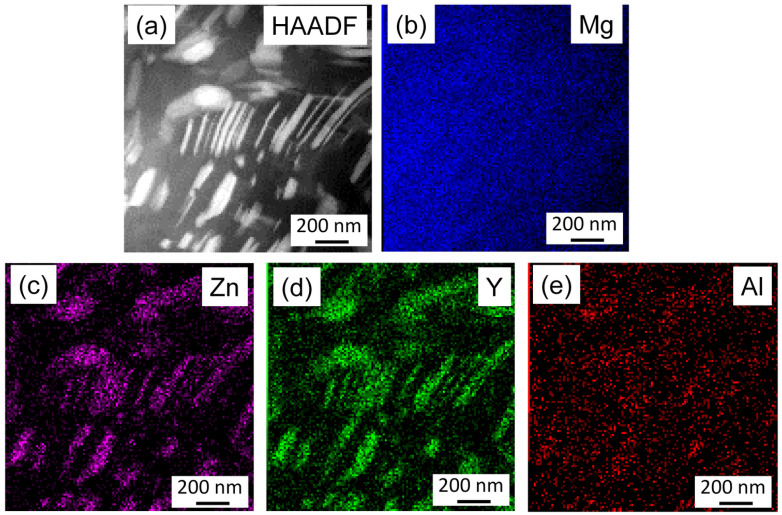
(**a**) HAADF image and (**b**–**e**) the corresponding Mg, Zn, Y, and Al element maps obtained by TEM-EDS for the initial sample.

**Figure 11:**
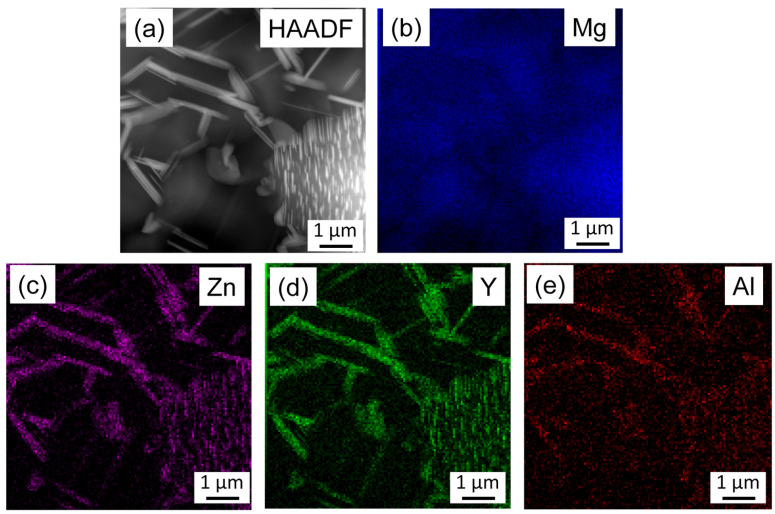
(**a**) HAADF image and (**b**–**e**) the corresponding Mg, Zn, Y, and Al element maps obtained by TEM-EDS for the annealed specimen.

Figure 10 shows an HAADF image and the corresponding Mg, Zn, Y, and Al element maps for the initial sample, as obtained by TEM-EDS. It is evident that the secondary-phase particles appearing with a bright contrast in the HAADF image are enriched in Zn and Y, and the Al concentration is also slightly higher in these particles compared to the matrix. These precipitates most probably correspond to the 18R Mg_12_YZn phase identified by XRD; however, the higher Al content suggests that Y and Zn are partly substituted by Al. Figure 11 shows an HAADF image and the corresponding Mg, Zn, Y, and Al element maps for the annealed specimen, as obtained by TEM-EDS. It can be seen that both the fine secondary-phase precipitates in the non-RX grains and the large lamellas in the RX grains are enriched in Zn, Y, and Al. Significant differences between the chemical compositions of these fine and coarse particles were not found.

**Figure 12:**
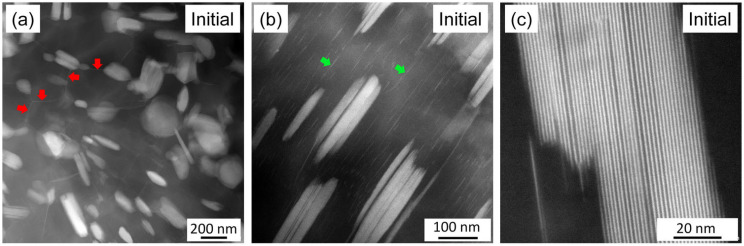
STEM images with different magnifications obtained on the initial sample. The red arrows in (**a**) indicate matrix grain boundaries enriched in heavy alloying elements as suggested by their lighter contrast compared to the grain interiors. The green arrows in (**b**) show individual CALs.

**Figure 13:**
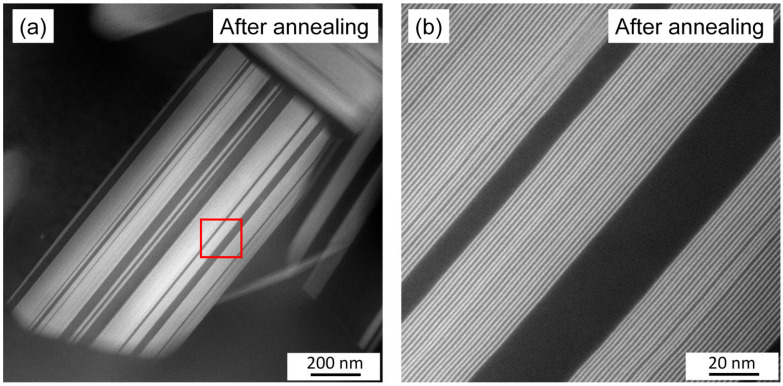
STEM micrographs taken on the annealed specimen. The area indicated by red square in (**a**) is shown in (**b**) at a higher magnification.

The STEM images in Figure 12 show the solute-enriched particles in the initial sample with different magnifications. The red arrows in Figure 12a indicate matrix grain boundaries enriched in heavy alloying elements, as suggested by their lighter contrast compared to the grain interiors. The micrograph in Figure 12b shows secondary-phase particles with a higher magnification. This image reveals that the secondary-phase particles developed in the form of lamellas. Additionally, individual CALs were also identified (highlighted by the green arrows). Figure 12c shows secondary-phase particles at a high magnification. Bright lines with a thickness of about 0.7–0.8 nm indicate solute-enriched basal planes. The spacing between the solute-enriched layers varies within grains, as illustrated in Figure 12c, suggesting that more than one polytype of LPSO structures coexist. A similar effect was observed in the coarse LPSO particles formed during annealing, as shown in Figure 13.

## 4. Discussion

Figure 3 reveals that the matrix lattice constants slightly deviated from the values obtained formerly for pure Mg. This effect was most probably caused by the solute elements in the matrix. The effects of solute Y and Zn on the lattice constants of Mg were different. Since the metallic atomic radii of Mg, Y and Zn are 160, 180, and 137 pm, respectively, the Y increased while the Zn decreased both lattice parameters *a* and *c* of the Mg matrix [28,29]. The alloy studied in this work also contained a low amount of Al, which has a similar radius (143 pm) as Zn, thus, Al also decreased the lattice parameters [30]. In the entire material, the concentration of Y was about twice of the sum of the Zn and Al contents. Furthermore, in the LPSO phase, the fractions of Y and Zn were close [13]; therefore, in the matrix, a higher amount of Y was expected compared to the sum of Zn and Al. In addition, for a unit solute concentration (1 at.%), the magnitude of the effect of Y on the change in the Mg lattice constants was shown to be higher (about 0.23% and 0.06% for lattice constants *a* and *c*, respectively) than that of Zn and Al (about −0.03% for both *a* and *c,* where the minus sign indicates a reduction in the lattice parameters) [28,29,30]. Thus, the matrix lattice constants of the present alloy were expected to be higher compared to pure Mg, which is in accordance with the experimental observation shown for the initial state in Figure 3. Both lattice parameters *a* and *c* were about 0.03–0.04% higher than those for pure Mg. It should be noted that SFs in SESFs may also provide contributions to the increase in the lattice constant *c*, as shown in a former study [31]. This additional effect can compensate for the lower influence of Y solutes on the lattice constant *c* compared to that of *a*, yielding a similar enhancement of the two lattice parameters in the present alloy before annealing.

During heating up to 833 K, the lattice parameter *a* of the matrix remained slightly higher than that of pure Mg (see Figure 3a). On the other hand, the lattice constant *c* of the matrix increased at a lower rate than that for pure Mg, mainly at high temperatures, as shown in Figure 3b. This phenomenon caused a slightly lower thermal expansion coefficient in the *c* direction (see Figure 4) and a decrease in the *c*/*a* ratio at high temperatures, as shown in Figure 5. The latter effect differed significantly in the trend valid for pure Mg (indicated by the blue curve in Figure 5). The relatively low lattice parameter *c* compared to pure Mg at high temperatures may have been caused by the reduction in the amount of SFs and the change in the matrix chemical composition during annealing. Namely, recrystallization can result in a disappearance of lattice defects, such as SFs, thereby eliminating their contribution to increasing the lattice constant *c*. In addition, partial melting at the temperature of 673 K and above can change the composition of the remaining solid material. Indeed, the strong decrease in the Mg peak intensity at 833 K (see Figure 6) suggests that about 47% of the material was melted. This value was obtained from the difference between the integrated intensity measured at the end of the heating (~175 count·deg) and the value predicted from the effect of the Debye–Waller factor (about 325 count·deg). The temperature of 833 K is significantly lower than the melting point of pure Mg (923 K), and the melting at this temperature is caused by the solute elements. The binary Mg-Y, Mg-Zn, and Mg-Al phase diagrams [32,33,34] suggest that the melted fraction should contain a higher concentration of alloying elements than the remaining solid, i.e., the chemical composition of the matrix probed by XRD may change due to the partial melting at 833 K, which can influence the lattice constants in accordance with the experimental results of this study. It should be noted that binary phase diagrams of Mg-Zn and Mg-Y systems suggest that the estimated volume fraction of the melting at 833 K (about one-half) can be achieved at Zn and Y concentrations of about 5–6 at.%. Such a local composition is certainly present in CALs, as well as in 10H, 18R, and 14H LPSO structures [13].

**Figure 14:**
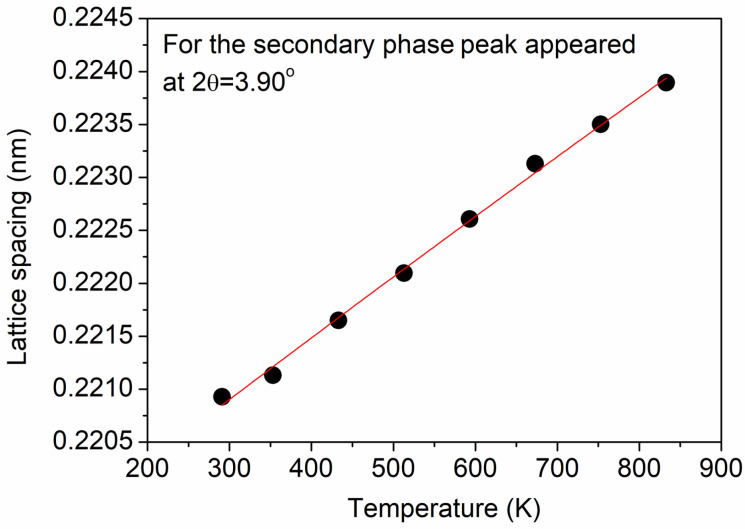
Change in the lattice spacing of the secondary-phase XRD peak appeared at about 2θ = 3.90° versus the temperature during heating. The error of the data is represented by the size of the symbols. The red curve corresponds to a quadratic polynomial fitted to the data points.

It is worth noting that, in addition to the thermal expansion of the matrix, the development of internal thermal stresses may also cause a change in the lattice constant when increasing the temperature. Namely, if the thermal expansion coefficients of the matrix and the secondary phase differ, stresses may develop in both the matrix and the precipitates when the temperature changes. For checking this effect, the thermal expansion coefficient of the secondary phase was estimated from the shift in the XRD peak appearing at about 2θ = 3.90° as a function of temperature. It was noted that the same peak of the secondary phase was used for the analysis of the variation in the fraction of this phase. Figure 14 shows the change in the interplanar spacing versus the temperature. The thermal expansion coefficient determined from this plot was (2.5 ± 0.1) × 10^−5^ K^−1^ for the whole temperature range between 293 and 833 K. This value characterizes the thermal expansion of the secondary phase perpendicular to the lattice planes reflecting the evaluated XRD peak. Between RT and 500 K, the estimated thermal expansion coefficient of the secondary phase was close to the corresponding values of the matrix in both the *a* and *c* directions (see Figure 4), suggesting that thermal stresses between the matrix and the secondary-phase particles did not develop. On the other hand, above 500 K, the thermal expansion coefficients of the matrix in both the *a* and *c* directions were significantly higher (3–4 × 10^−5^ K^−1^) than that for the precipitates (2.5 × 10^−5^ K^−1^). Therefore, most probably, internal stresses develop at the interfaces of the matrix and the secondary-phase particles during annealing at high temperatures. This effect may contribute to the driving force of melting with an increasing temperature.

Figure 6 shows that neither recrystallization nor partial melting during heating considerably influenced the secondary-phase fraction. On the other hand, immediately at the beginning of cooling between 833 and 753 K, the XRD intensity of the secondary phase increased by about twice, and this value remained constant until the end of cooling. This effect might have been caused by the increase in the amount of the solute-enriched secondary phase due to the joining of additional solute atoms to the secondary phase. This scenario is less probable, since the quantitative evaluation of Figure 8a,b did not show the change in the secondary-phase fraction during annealing (for both states, it was about 20%). Rather, most probably an improvement in the regularity of the spatial distribution of the FCC units in the CAL layers and/or the periodicity of the CALs and Mg layers occurred, which resulted in an increase in the XRD intensity of the solute-enriched secondary phase. However, this mechanism requires further confirmation by atomic-resolution TEM, which is beyond the scope of the present paper. It should be noted that, according to the STEM images presented in Figure 12 and Figure 13, the individual CALs disappeared from the grain interiors during annealing, which suggests that the solute atoms in the matrix did not cluster in the CALs during cooling.

**Figure 15:**
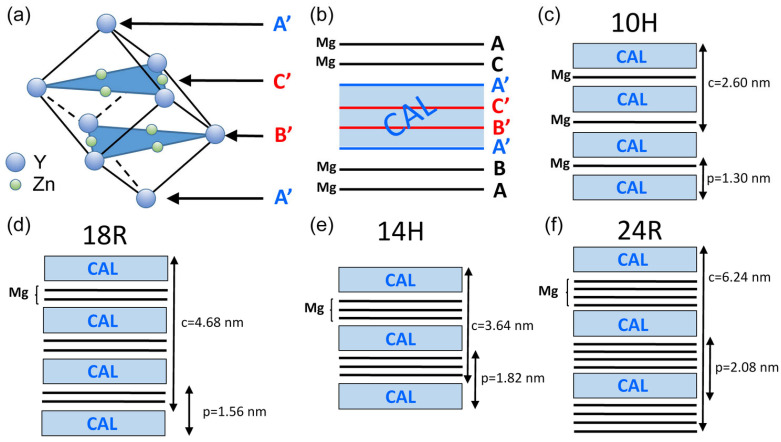
Schematics showing (**a**) FCC ordering of Y and Zn atoms in CALs and (**b**) the stacking of the close-packed planes in the close vicinity of a CAL. The capital letters A, B, and C indicate the three different close-packed plane positions in the structure. The prime at the capital letters indicates solutes in the close-packed planes. The planes marked by red letters contain both Y and Zn, while the blue letters correspond to planes containing only Y beside Mg. The pure Mg planes are indicated by black letters in (**b**). Figures (**c**–**f**) show 10H, 18R, 14H, and 24R LPSO structures, respectively. These schematics also contain the characteristic length dimensions of these LPSO structures perpendicular to the close-packed planes. *c*—lattice constant perpendicular to the close-packed planes. *p*—periodicity of the variation in CALs and pure Mg planes.

It is worth noting that the LPSO structure of the solute-enriched particles was not perfectly ordered either before or after annealing, as suggested by Figure 12 and Figure 13. Namely, while the bright secondary-phase particles seemed to be uniform in the lower-resolution STEM images (see Figure 12a), the micrographs with a higher resolution revealed that these particles consisted of narrower lamellas, as shown in Figure 12b. The clustering nature of the solute-rich secondary phase lamellas was also characteristic for the annealed specimen (see Figure 9b and Figure 12a). Furthermore, the structure inside the individual lamellas was not uniform. This means that the spacing between the bright solute-enriched layers varied, which suggests the co-existence of different LPSO structures in the same lamella. The bright layers were most probably CALs with the FCC sequence A’B’C’A’, as suggested by former studies on Mg-LPSO materials [10,11,13]. The FCC cell of the CAL structure is shown schematically in Figure 15a. The two middle close-packed planes (red B’ and C’) in the CALs contained both Y and Zn atoms, while the two outer planes (blue A’) contained only Y beside Mg. The CALs were separated by pure Mg close-packed planes, as shown in Figure 15b.

Figure 15c–f show schematically the four different LPSO structures. The numbers of the pure Mg close-packed planes between the CALs are one, two, three, and four for the 10H, 18R, 14H, and 24R LPSO polytypes, respectively [13]. The spatial arrangement of the solute-enriched FCC clusters in the consecutive CALs varied; therefore, the lattice constant perpendicular to the close-packed planes (denoted as *c*) differed from the periodicity of the solute-enriched and pure Mg layers marked with *p* in Figure 15. The value of *p* can be easily observed in the STEM micrographs and is a characteristic feature of the polytypes. Figure 15 shows that the values of this periodicity for the 10H, 18R, 14H, and 24R LPSO structures were 1.30, 1.56, 1.82, and 2.08 nm, respectively. In the STEM images taken in this study, the periodicity of the bright-dark layer couples varied between 1.48 ± 0.02 and 2.03 ± 0.02 nm in the initial sample, while for the annealed sample, this range was 1.51–2.10 nm (error: ±0.02 nm), as illustrated in Figure 12c and Figure 13b, respectively. These ranges suggest the co-existence of the 18R, 14H, and 24R LPSO polytypes in the secondary-phase grains both in the initial state and after annealing. This observation is in accordance with former studies [13,35].

In summary, our study revealed that there were reversible and irreversible phenomena in the investigated Mg-Y-Zn-Al alloy processed by the RSRC technique and subsequently annealed up to the temperature of 833 K. Namely, the amount and the average lattice constants of the matrix behaved reversibly during heating and cooling, as suggested by Figure 2 and Figure 6. On the other hand, the XRD intensity of the secondary phase increased in an irreversible way during cooling, as shown in Figure 6. Considering these observations, the following scenario can be established. During heating, recrystallization occurred together with the disappearance of lattice defects such as SESFs. In addition, between 673 and 833 K, the material was partially melted in those volumes where (i) there was a higher solute concentration in the Mg matrix, (ii) the periodicity *p* was lower in the solute-enriched phase associated with a higher alloying element content, (iii) the density of defects (SFs and dislocations) was higher, (iv) the size of the Mg grains and/or the solute-enriched particles was smaller, and (v) internal thermal stresses developed. These five effects facilitated the melting of the material locally. In the remaining solid material, the *c*/*a* lattice constant ratio was lower due to the change in the chemical composition. This effect was most pronounced at 753 and 833 K. During cooling, the relative intensity of the secondary phase was irreversibly increased, which was most probably caused by the formation of a more ordered solute-enriched secondary phase structure in the grains crystallized from the melt.

Mg-LPSO alloys usually exhibit an excellent mechanical performance, including a high strength at both room and elevated temperatures, as well as a high strain rate superplasticity. Therefore, these materials have great potential for use as structural components in transportation vehicles (cars, trains, and ships), whose weight reduction is essential for reducing their ecological footprint. The outstanding strength-to-weight ratio of Mg-LPSO alloys is caused mainly by solute-enriched CALs and LPSO particles. The thermal stability of these items of the microstructure significantly influences the mechanical behavior of these alloys. Therefore, the results of this study are of great importance, since they reveal the evolution of these solute-enriched microstructural features. The dissolution of CALs during heating, as well as the coarsening and ordering of the LPSO phase observed in this study, most probably significantly influence the mechanical behavior of the alloys. In addition, the detected change in the *c*/*a* lattice parameter ratio of the matrix at high temperatures can have an effect on the active dislocation slip systems, which influence the deformability of the material. Further studies are planned to be performed in order to reveal the effect of annealing on the mechanical performance of this RSRC-processed dilute Mg-Y-Zn-Al alloy.

## 5. Conclusions

A Mg—0.9%, Zn—2.05%, Y—0.15% Al (at%) alloy processed by the RSRC technique was heated at a rate of 0.66 K/s up to 833 K, and the structure evolution in the matrix and the secondary phase were studied. The following conclusions were drawn from the results:

1. Before annealing, both lattice constants of the Mg matrix were slightly higher than those of pure Mg, which can be attributed to the higher concentration of Y compared to Zn, and the effect of SFs in CALs. During heating at temperatures higher than 753 K, the rate of increase in the lattice parameter *c* was lower than that for the lattice constant *a*, resulting in a decrease in the ratio of *c*/*a*. This phenomenon can be attributed to the decrease in the amount of SFs due to recrystallization and the change in the solute content owing to partial melting. The lattice constant change was reversible during heating and cooling. The thermal expansion coefficient of the matrix before annealing in both the *a* and *c* directions was about 2.5 × 10^−5^ K^−1^, which increased when increasing the temperature. On the other hand, the thermal expansion coefficient of the secondary phase remained at ~2.5 × 10^−5^ K^−1^ in the whole studied temperature range. Therefore, the significant difference between the thermal expansion coefficients of the matrix and the secondary phase above 500 K may cause internal stresses at the interfaces.
2. During heating, the secondary-phase fraction did not change. On the other hand, when cooling started, the XRD integral intensity of the secondary phase increased, suggesting the formation of a more ordered solute-enriched secondary-phase structure in the crystallized grains. It should be noted, however, that both before and after annealing, different LPSO polytypes coexisted in the secondary-phase particles. During annealing, both the matrix grains and the solute-enriched particles were coarsened. The changes in the morphology and the XRD intensity of the secondary phase were irreversible processes during annealing.

The performed research contributes to the understanding of the high-temperature thermal stability of the microstructure in RSRC-processed dilute Mg-Y-Zn-Al alloys, which is essential for developing novel Mg alloys with enhanced properties in extreme conditions, e.g., for high-temperature applications. The observed changes in the lattice parameters and the solute-enriched CALs and LPSO particles during annealing can have a significant effect on the mechanical behavior at elevated temperatures, which may be a topic of further research.

## Figures and Tables

**Table 1:** The fraction of RX region and the average grain sizes in the RX area in the initial alloy and the annealed sample, as obtained by EBSD. The values in the parentheses after the average grain size give the standard deviation of the size distribution.

Sample	RX Fraction (%)	Average Size of RX Grains (μm)
Initial	62	0.63 (0.28)
After annealing	97	2.80 (1.20)

## Data Availability

The raw data supporting the conclusions of this article will be made available by the authors on request.
